# Late-life, visit-to-visit blood pressure variability and its association with sex-specific long-term cognitive outcomes

**DOI:** 10.1038/s41371-025-01087-5

**Published:** 2025-10-18

**Authors:** Michael E. Ernst, Kerry M. Sheets, Katherine L. Webb, Michelle A. Fravel, Robyn L. Woods, Lawrence Beilin, Suzanne G. Orchard, Raj C. Shah, Kevan R. Polkinghorne, Christopher M. Reid, Rory Wolfe, Anne Murray, Paul Lacaze, Joanne Ryan

**Affiliations:** 1 Department of Pharmacy Practice and Science, College of Pharmacy; The University of Iowa, Iowa City, IA USA; 2 Department of Family and Community Medicine, Carver College of Medicine; The University of Iowa, Iowa City, IA USA; 3 Department of Medicine, Hennepin Healthcare, Minneapolis, MN USA; 4 Department of Medicine, University of Minnesota, Minneapolis, MN USA; 5 School of Public Health and Preventive Medicine, Monash University, Melbourne, VIC Australia; 6 Medical School Royal Perth Hospital, University of Western Australia, Perth, WA Australia; 7 Department of Family Medicine and Rush Alzheimer’s Disease Center, Rush University Medical Center, Chicago, Ill USA; 8 Department of Nephrology, Monash Medical Centre, Monash Health; and, Department of Medicine, Monash University, Melbourne, VIC Australia; 9 School of Public Health, Curtin University, Perth, WA Australia; 10 Berman Center for Outcomes and Clinical Research, Hennepin Health Research Institute, Minneapolis, MN USA

**Keywords:** Risk factors, Ageing

## Abstract

In 19 114 community-dwelling older adults enrolled in the ASPREE trial, high late-life visit-to-visit blood pressure variability (BPV) was associated with increased risks of incident dementia and cognitive decline during the median 4.7 year trial period. Whether sex or apolipoprotein E (ApoE) affect these associations over longer-term follow-up is unknown. We investigated the association between BPV and long term risks of dementia and cognitive decline using data from the ASPREE-eXTension (ASPREE-XT) study (median 8.3 year follow-up for dementia, 7.3 years for cognitive decline, after BPV estimation). Long-term BPV was estimated using standard deviation (SD) of the mean systolic BPs measured at ASPREE baseline, year 1 and 2 visits. Incident dementia was an adjudicated secondary endpoint and cognitive decline was defined as ≥1.5 SD decline in score from baseline sustained on the same of one, or more, standardized cognitive tests administered annually/biennially throughout ASPREE/ASPREE-XT. Analyses were stratified a priori by sex, using sex-specific SD tertiles of systolic BPV. Multivariable Cox proportional hazards regression comparing the highest BPV tertile to the lowest showed increased risk of dementia (HR = 1.33, 1.10–1.61) and cognitive decline (HR = 1.17, 1.06–1.30) in males, and cognitive decline in females (HR = 1.17, 1.07–1.28). In ApoE genotyped participants (81%), females in the highest BPV tertile lacking the ɛ4 allele had increased risk of dementia (HR = 1.39; 1.04–1.84), while risk of cognitive decline was increased in both sexes lacking the ɛ4 allele (males HR = 1.25; 1.09–1.43; females HR = 1.14; 1.01–1.29). These findings suggest both sex and ApoE impact the association of high BPV with long-term cognitive changes.

## Introduction

The global incidence of dementia continues to accelerate, with recent estimates suggesting an increase of 117% from 1990 to 2016 [1]. Decreases in fertility combined with increases in life expectancy ensure the upward trend for more people living at the oldest ages will persist, and the number of persons with dementia will nearly triple in the coming decades, from 57.4 million cases in 2019 to an estimated 152.8 million cases in 2050 [2, 3]. There are several notable risk factors for worsening brain health with aging, and one of the strongest is that of elevated blood pressure (BP) [4]. The risk of dementia is 42% higher in persons with untreated hypertension, positioning BP as a central focus for a readily modifiable intervention to protect cognitive function [5].

In recent years, high variability in BP over time, across multiple visits, has emerged as a risk factor for dementia and cognitive decline. This risk appears to exist independent of the mean BP upon which treatment decisions in routine care are normally based [6, 7]. Most prior studies have been conducted in middle-aged populations with established hypertension or uncontrolled BP [8], or in hypertension intervention trials [9]. Others were limited by single cognitive assessments [10], and short follow-up periods [7], all of which may distort real-world predictions of the long-term impact of BP variability in those reaching older ages with intact cognition. Furthermore, few studies have investigated potential sex-related differences in the relationship of BPV with cognition, as well as the interplay of potent cognitive risk factors, such as the presence of the apolipoprotein E (ApoE) ɛ4 allele [11].

In the ASPirin in Reducing Events in the Elderly (ASPREE) trial, we previously demonstrated that participants with high levels of systolic BP variability (BPV) across multiple study visits early in the trial were at higher risk of incident dementia and cognitive decline during the in-trial follow-up period, relative to those with lower systolic BP variability [7]. Sex is an important biological variable, with accumulating evidence suggesting that the different cardiovascular risk profiles of males and females leads to heterogeneity between the sexes in the outcomes associated with different exposures [12]. Our earlier analysis revealed the relationship between BPV and dementia was modified by sex, appearing stronger for males than females, but was limited by a short follow-up time (median 2.7 years for dementia, 2.0 years for cognitive decline) after excluding the time window of BPV estimation [7]. The in-trial and post-trial observational periods of a long-term study such as ASPREE can be analyzed as a longitudinal cohort study, providing additional precision in risk factor evaluation. In this analysis, we extend our in-trial findings by including up to 6 additional years of follow-up per participant, to further ascertain how variability in BP across multiple annual study visits impacts the long-term risk of incident dementia and cognitive decline between the sexes, as well as how ApoE ɛ4 carrier status affects these risks.

## Materials and methods

### Study cohort

This report is a post-hoc analysis of data from the ASPREE and ASPREE-eXTension (ASPREE-XT) studies [13, 14]. Briefly, from 2010–2014, 19 114 community-dwelling adults in Australia and the US aged 70 years and older (65 years and older if a US minority) were enrolled into the ASPREE randomized, placebo-controlled trial of 100 mg daily aspirin as a preventive agent to prolong disability-free survival (a composite of survival free of dementia or significant physical disability). As a condition of enrollment, individuals had to be free of documented evidence of cardiovascular disease, dementia or significant cognitive deficit (a Modified Mini-Mental State Examination [3MS] score of less than 78/100), or independence-limiting physical disability, uncontrolled BP (systolic BP ≥ 180 mmHg and/or diastolic BP ≥ 105 mmHg), or any concurrent illness expected to limit survival to less than five years.

The intervention phase of the ASPREE trial was completed in 2017 after a median of 4.7 years of follow-up, finding no significant benefit of aspirin but increased bleeding risk [13]. Surviving participants were then invited to continue their annual follow-up visits in a long-term observational follow-up study (ASPREE-XT) [14]. Of the original ASPREE cohort, 83% of participants continued into the observational extension, with follow-up totaling up to 13 years for those enrolled during the earliest period of the trial [14]. Written informed consent was obtained from all participants for ASPREE and re-consent was obtained for ASPREE-XT. Both studies were approved by institutional review boards in both countries. The ASPREE study is registered at ClinicalTrials.gov (NCT01038583) and at ISRCTN83772183.

### Assessment of long-term, visit-to-visit BP variability (BPV)

At baseline and each annual study visit during ASPREE, standardized BP measurements were obtained in each participant in triplicate, using a validated oscillometric device, with at least one minute between readings, according to American Heart Association guidelines [15]. The mean of the three readings was recorded as the BP for that visit. We estimated the long-term, visit-to-visit BPV using the within-individual standard deviation (SD) of the mean systolic BP obtained from the baseline, first and second annual visits of ASPREE (three visits in total, spanning 2 years), consistent with prior analyses [7, 16–18]. Complete-case analysis was used for the BPV estimation.

### Cognitive battery

Cognition was assessed longitudinally throughout ASPREE and ASPREE-XT using a standardized cognitive battery which included the 3MS for global cognition [19], the Hopkins Verbal Learning Test-Revised (HVLT-R) for delayed episodic memory [20], the single letter (F) Controlled Oral Word Association Test (COWAT) for verbal fluency [21], and the Symbol Digit Modalities Test (SDMT) for processing speed and attention [22]. This battery was administered at the baseline and Year 1 study visits, biennially during follow-up visits (Year 3, 5, and 7, and close-out of ASPREE), and then annually during ASPREE-XT. The Center for Epidemiologic Studies-Depression (CES-D) scale [23] was administered prior to the cognitive battery to account for the possible confounding effect of depression on cognitive function.

### Outcomes

Incident dementia was a pre-specified endpoint of ASPREE and ASPREE-XT, and adjudicated using the DSM-IV criteria by an expert panel of clinicians, blinded to treatment arm [24]. Briefly, a 3MS score <78/100 or a decline of >10.15 points from a 5-year predicted score (adjusted for age and education), a medical record report of dementia or memory problems, or a prescription for cholinesterase inhibitor, triggered additional cognitive testing scheduled at least 6 weeks after the trigger (to eliminate effects of potential delirium). The additional testing included the Alzheimer’s Disease Assessment Scale – Cognitive subscale [25], Color Trails [26], Lurian overlapping figures [27], and the Alzheimer Disease Cooperative Study Activities of Daily Living Scale [28]. Clinical case notes and other supporting documentation were additionally obtained for these dementia triggers and submitted to the endpoint adjudicators.

A sustained ≥1.5 SD decline from a participant’s baseline score on any of the cognitive tests (3MS, SDMT, HVLT-R delayed recall and/or COWAT) during follow-up was used to identify the non-prespecified endpoint of incident cognitive decline (CIND) [24]. The decline must have occurred for at least two consecutive testing timepoints on the same cognitive test. This outcome was established since participants could experience clinically noticeable declines in cognitive function tests but fail to meet the threshold required in the protocol to trigger additional dementia assessment testing.

### Statistical analysis

Analyses were stratified a priori by sex. Tertiles of standard deviation (SD) of systolic BPV were initially defined for the cohort, separately for males and females. The risk of dementia according to tertiles of BPV, as well as BPV used as a continuous variable, beginning at the end of the BPV estimation window (post-Year 2 annual visit) was calculated using time-to-event Cox proportional hazards regression models, with tertile 1 as the reference. Participants who met the dementia endpoint during the BPV estimation period were excluded from the analyses to avoid immortal time bias, whereas all other participants were at risk (not excluded) for the dementia outcome as long as they had follow-up after the BPV estimation period. Hazard ratios (HR) and corresponding 95% confidence intervals (CI) are reported. Initial age-adjusted models (Model 1) were further adjusted for baseline variables including ethnicity, education, depression, diabetes, dyslipidemia, body mass index, smoking, alcohol, 3MS, and living situation (Model 2), followed by additional adjustments for average systolic BP from baseline – year 2 (the BPV estimation window) and use of antihypertensive medications at baseline (Model 3). For covariates in models 2 and 3, only BMI (n = 68, 0.41%) and depression (n = 3, 0.02%) had missing values and were imputed using sex- and age-adjusted means.

The models were repeated, with stratification by ApoE ɛ4 carrier status. Biospecimen collection, DNA extraction, genome-wide genotyping and determination of ApoE status in ASPREE is described previously [29, 30]. ApoE ɛ4 carrier was defined as having ≥1 ApoE ɛ4 allele (e.g., ApoE ɛ3/ɛ4, ApoE ɛ4/ɛ4, ApoE ɛ1/ɛ4, ApoE ɛ1/ɛ3:ɛ2/ɛ4).

We conducted sensitivity analyses to confirm the main findings. These included expanding the time period of BPV estimation from the original three, to four visits (i.e., baseline, annual study visits Year 1, 2, and 3), and recalculating the risk of dementia with Year 3 as time zero. From our prior analyses, SD and other indices of BPV were highly correlated, with absolute real variability (ARV) having the least correlation [7, 16–18]. Therefore, we also used ARV as an alternate estimate of BPV, and repeated our primary analysis.

Analyses to determine the risk of CIND were conducted identically to dementia, with cognitive decline replacing dementia as the outcome and excluding those who met the CIND endpoint during the BPV estimation period. To further investigate the relationship of BPV on specific cognitive domains, linear mixed models were used to examine longitudinal changes in the individual cognitive tests. Linear mixed models were used rather than treating CIND as a binary endpoint as these models allow for missing data due to the individual timing of the tests and gaps in time points. Models included fixed effects for time and BPV (i.e., providing an estimate of the cross-sectional association of BPV with the cognitive test score), and a time-BPV interaction term (i.e., providing an estimate of the association of BPV with longitudinal change in the cognitive test score) with a random intercept for each individual. P-values of less than 0.05 (two-sided) were used as the cutoff for statistical significance. Analyses were conducted using R version 4.3.3 (R Core Team, 2024).

## Results

**Fig. 1:**
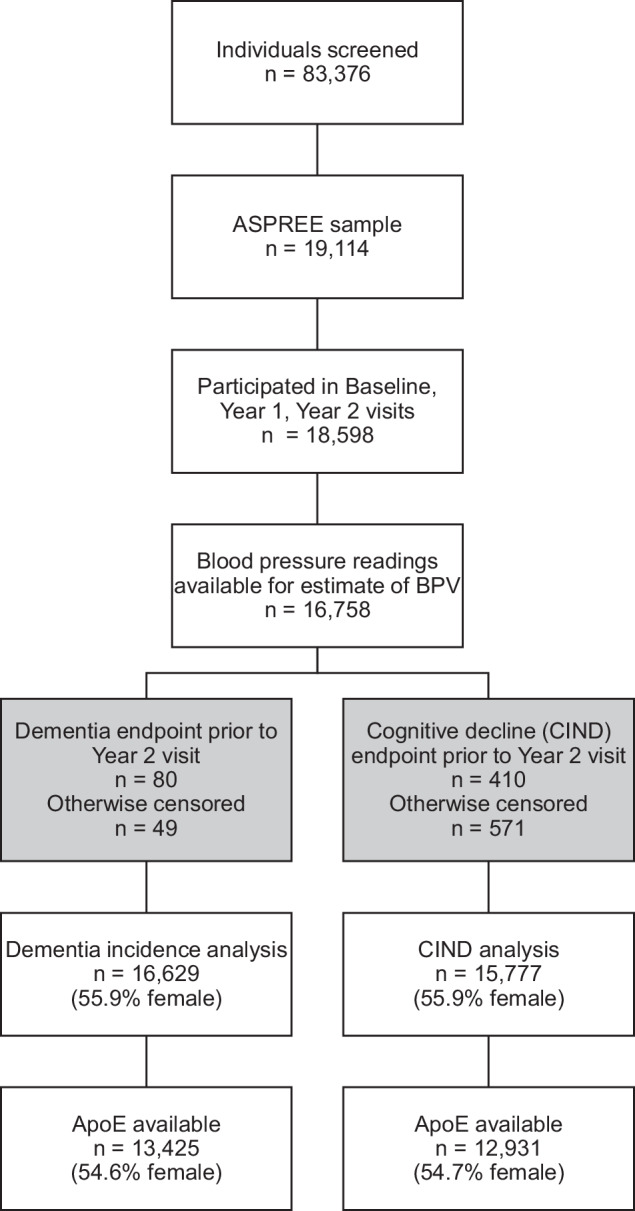
CONSORT flow diagram of participants included in the analysis.

The analytic cohort for incident dementia consisted of 16 629 individuals (7 335 males, 9 294 females) who were free of dementia at the Year 2 visit and had BP measured at baseline and annual study visit Year 1 and 2, enabling estimation of long-term systolic BPV (Fig. 1). For CIND, the cohort was slightly smaller (N = 15 777). The mean BP at baseline increased across SD tertiles in both males and females (Table 1), as did the prevalence of antihypertensive use, and the average systolic BP during the BPV estimation period. A higher proportion of participants in the highest SD tertile were 74 years of age or older, and comorbidities such as diabetes and chronic kidney disease were more common in those participants in the highest tertile of BPV. The mean 3MS cognitive score was lower in participants in the highest SD tertile of BPV. Similar demographic patterns were observed for the CIND cohort (data not shown).

**Table 1:** Baseline characteristics of ASPREE participants included in the incident dementia analysis, by sex and tertiles (T) of standard deviation of systolic blood pressure variability.

	Males, n = 7335			Females, n = 9294		
	T1, n = 2508	T2, n = 2404	T3, n = 2423	T1, n = 3121	T2, n = 3086	T3, n = 3087
**Standard deviation of SBP, mean (SD)**	4.23 (1.62)	9.03 (1.36)	16.56 (4.40)	4.44 (1.70)	9.39 (1.42)	17.36 (4.95)
**Standard deviation of SBP, range**	0–6.66	6.81–11.53	11.55–41.74	0–7.02	7.09–12.06	12.10–57.71
**Baseline SBP, mmHg, mean (SD)**	137.91 (13.77)	140.46 (14.82)	145.05 (17.78)	133.56 (14.48)	136.42 (15.60)	142.52 (18.67)
**Baseline DBP, mmHg, mean (SD)**	77.19 (8.94)	77.86 (9.33)	79.17 (10.26)	75.33 (9.51)	76.27 (9.81)	78.22 (11.04)
**Baseline antihypertensive medications, n (%)**	1119 (44.6)	1092 (45.4)	1370 (56.5)	1521 (48.7)	1678 (54.4)	1892 (61.3)
**Average SBP over BPV estimation period, mean (SD)**	137.72 (13.36)	139.43 (13.10)	141.90 (12.99)	133.29 (13.99)	135.56 (13.63)	139.65 (13.51)
**ApoE genotype, n (%)**						
ε4 allele present	556 (22.2)	538 (22.4)	516 (21.3)	605 (19.4)	631 (20.4)	596 (19.3)
ε4 allele not present	1544 (61.6)	1468 (61.1)	1470 (60.7)	1902 (60.9)	1805 (58.5)	1794 (58.1)
APOE genotype not available	408 (16.3)	398 (16.6)	437 (18.0)	614 (19.7)	650 (21.1)	697 (22.6)
**Ethno-racial group, n (%)**						
Australian white	2245 (89.5)	2153 (89.6)	2148 (88.7)	2670 (85.5)	2619 (84.9)	2595 (84.1)
U.S. white	114 (4.5)	102 (4.2)	87 (3.6)	204 (6.5)	225 (7.3)	208 (6.7)
African-American	69 (2.8)	66 (2.7)	71 (2.9)	131 (4.2)	143 (4.6)	169 (5.5)
Hispanic/Latino	52 (2.1)	43 (1.8)	61 (2.5)	82 (2.6)	67 (2.2)	70 (2.3)
Other	28 (1.1)	40 (1.7)	56 (2.3)	34 (1.1)	32 (1.0)	45 (1.5)
**Age, y, n (%)**						
65–73	1367 (54.5)	1265 (52.6)	1177 (48.6)	1703 (54.6)	1542 (50.0)	1396 (45.2)
≥74	1141 (45.5)	1139 (47.4)	1246 (51.4)	1418 (45.4)	1544 (50.0)	1691 (54.8)
**Education y, n (%)**						
<12	1099 (43.8)	1035 (43.1)	1042 (43.0)	1464 (46.9)	1387 (44.9)	1434 (46.5)
12+	1409 (56.2)	1369 (56.9)	1381 (57.0)	1657 (53.1)	1699 (55.1)	1652 (53.5)
**Alcohol use, n (%)**						
Current	2099 (83.7)	2046 (85.1)	2022 (83.5)	2266 (72.6)	2231 (72.3)	2229 (72.2)
Former	174 (6.9)	154 (6.4)	167 (6.9)	150 (4.8)	122 (4.0)	150 (4.9)
Never	235 (9.4)	204 (8.5)	234 (9.7)	705 (22.6)	733 (23.8)	708 (22.9)
**Body Mass Index, kg/m**^**2**^**, mean (SD)**	27.93 (3.85)	27.94 (3.87)	28.12 (3.97)	28.05 (4.98)	28.05 (5.22)	28.40 (5.34)
**Living alone, n (%)**	482 (19.2)	458 (19.1)	519 (21.4)	1246 (39.9)	1276 (41.4)	1326 (43.0)
**Current or past smoker, n (%)**	1376 (54.9)	1332 (55.4)	1423 (58.7)	1080 (34.6)	1041 (33.7)	1068 (34.6)
**Diabetes, n (%)**	266 (10.6)	291 (12.1)	332 (13.7)	270 (8.7)	247 (8.0)	304 (9.8)
**Depressive Symptoms, n (%)**	181 (7.2)	178 (7.4)	181 (7.5)	358 (11.5)	320 (10.4)	348 (11.3)
**Dyslipidemia, n (%)**	1393 (55.5)	1328 (55.2)	1331 (54.9)	2280 (73.1)	2235 (72.4)	2285 (74.0)
**Chronic Kidney Disease, n (%)**	508 (21.9)	546 (24.4)	648 (28.7)	708 (24.3)	702 (24.4)	877 (30.5)
**Hypertension, n (%)**	1712 (68.3)	1769 (73.6)	2025 (83.6)	1983 (63.5)	2233 (72.4)	2571 (83.3)
**Anti-hypertension medications, n (%)** (not mutually exclusive)						
ACE inhibitors	446 (17.8)	402 (16.7)	542 (22.4)	452 (14.5)	501 (16.2)	590 (19.1)
ARBs	492 (19.6)	550 (22.9)	677 (27.9)	764 (24.5)	871 (28.2)	1033 (33.5)
CCBs	421 (16.8)	400 (16.6)	465 (19.2)	505 (16.2)	571 (18.5)	627 (20.3)
Diuretics	333 (13.3)	324 (13.5)	444 (18.3)	631 (20.2)	659 (21.4)	769 (24.9)
**Statin medications, n (%)**	712 (28.4)	663 (27.6)	680 (28.1)	1028 (32.9)	1010 (32.7)	1084 (35.1)
**Aspirin treatment assignment, n (%)**	1251 (49.9)	1188 (49.4)	1193 (49.2)	1574 (50.4)	1517 (49.2)	1525 (49.4)
**Pulse Pressure, mean (SD)**	60.73 (12.08)	62.60 (12.37)	65.88 (14.25)	58.22 (12.89)	60.15 (13.65)	64.29 (15.23)
**Heart Rate, mean (SD)**	69.22 (10.36)	68.97 (11.01)	68.52 (11.13)	72.54 (9.85)	72.02 (10.21)	71.19 (10.64)
**Standard deviation of heart rate variability, mean (SD)**	5.36 (3.47)	5.67 (3.89)	6.16 (4.23)	5.04 (3.41)	5.25 (3.64)	5.66 (3.80)
**Cognitive performance**						
3MS, mean (SD)	93.18 (4.49)	92.77 (4.74)	92.85 (4.59)	94.29 (4.30)	94.22 (4.21)	94.21 (4.34)
HVLT-R delayed recall, mean (SD)	7.32 (2.76)	7.30 (2.86)	7.24 (2.81)	8.31 (2.66)	8.35 (2.65)	8.31 (2.65)
SDMT, mean (SD)	36.41 (9.62)	35.89 (9.85)	35.11 (9.94)	38.77 (9.80)	38.67 (9.99)	37.54 (10.09)
COWAT, mean (SD)	11.68 (4.48)	11.48 (4.47)	11.55 (4.48)	12.62 (4.64)	12.74 (4.46)	12.65 (4.54)

Diabetes defined as a self-report, fasting glucose ≥126 mg/dl, or receiving pharmacologic treatment for diabetes (regardless of fasting glucose level). Depressive symptoms defined as a 10-item Center for Epidemiological Studies Depression Scale [23] score of ≥8. Dyslipidemia defined as serum cholesterol level of ≥212 mg per deciliter (≥5.5 mmol per liter) in Australia and ≥240 mg per deciliter ( ≥ 6.2 mmol per liter) in the United States, low-density lipoprotein level of >160 mg per deciliter (>4.1 mmol per liter), or use of a cholesterol-lowering medication. Chronic kidney disease defined as estimated glomerular filtration rate <60 mL/min per 1.73 m2 or albumin to creatinine ratio ≥3 mg/mmol. Hypertension defined as SBP ≥ 140 mmHg, DBP ≥ 90 mmHg, or receiving treatment for high BP (regardless of BP level).

*SBP* systolic blood pressure, *DBP* diastolic blood pressure, *T* tertile of systolic blood pressure variability, *ApoE* apolipoprotein E., *3MS* modified mini-mental state examination [19], *HVLT-R* Hopkins verbal learning test-revised [20], *SDMT* symbol digit modalities test [22], *COMT* controlled oral word association test [21].

### Incident dementia

**Fig. 2:**
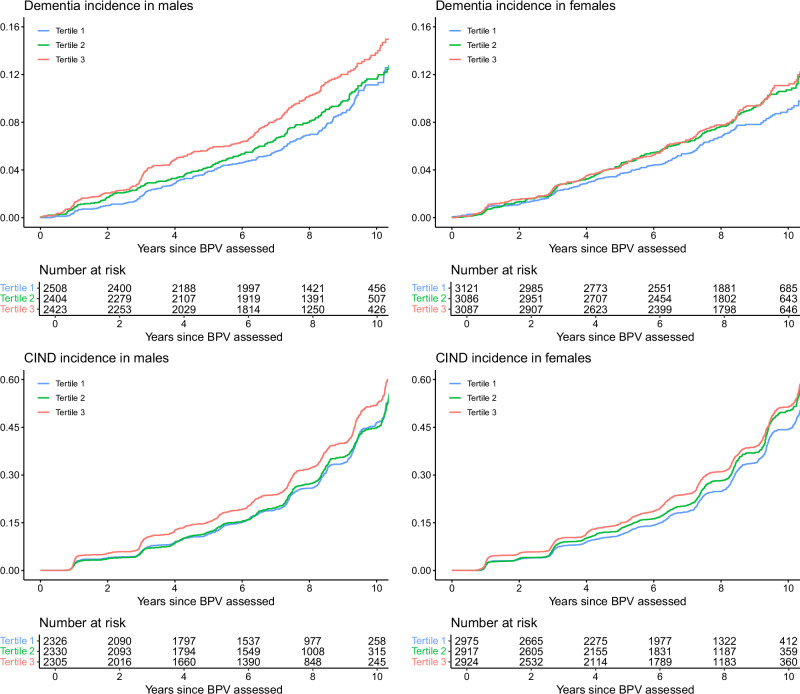
Sex-specific cumulative incidences of adjudicated dementia and cognitive decline according to tertile of blood pressure variability. **Panel A**: Cumulative incidence of dementia. BPV blood pressure variability. **Panel B**: Cumulative indicence of cognitive decline (CIND). BPV blood pressure variability.

During a median follow-up of 8.3 years after the BPV estimation period, 660 adjudicated incident dementia cases occurred in males and 739 in females (Fig. 2). For both sexes, there was a progressive increase in the number of dementia cases with increasing BPV tertile. Table 2 shows the fully-adjusted Cox proportional hazards model for the association between BPV and incident dementia. When BPV was examined as a continuous variable, the HR for incident dementia in males was 1.09 (95% CI = 1.02, 1.16; p = 0.009), whereas the relationship was not statistically significant in females (HR = 1.03; 95% CI = 0.97, 1.09; p = 0.390). The HR for incident dementia in males in SD tertile 3 compared to SD tertile 1 was 1.33 (95% CI = 1.10, 1.61; p = 0.003), while in females, the HR was 1.15 (95% CI = 0.96, 1.38; p = 0.132).

**Table 2:** Cox proportional hazards analysis for the association between systolic blood pressure variability (BPV) and incident dementia and cognitive decline (CIND), according to sex.

	Males					Females				
	N	# of events	Event Rate per 1000 person-years	HR (95% CI)	p-value	N	# of events	Event Rate per 1000 person-years	HR (95% CI)	p-value
**Dementia**										
BPV (continuous, per 5 mmHg)	7335	660	11.8	1.09 (1.02, 1.16)	0.009	9294	739	10.2	1.03 (0.97, 1.09)	0.390
BPV Tertile 1	2508	197	10.2	Ref.		3121	224	9.1	Ref.	
BPV Tertile 2	2404	214	11.5	1.06 (0.87, 1.29)	0.548	3086	256	10.7	1.14 (0.96, 1.37)	0.142
BPV Tertile 3	2423	249	14.0	1.33 (1.10, 1.61)	0.003	3087	259	10.9	1.15 (0.96, 1.38)	0.132
**Cognitive decline (CIND)**										
BPV (continuous, per 5 mmHg)	6961	2240	49.0	1.06 (1.03, 1.1)	p < 0.001	8816	2881	49.9	1.05 (1.02, 1.08)	0.001
BPV Tertile 1	2326	700	45.1	Ref.		2975	897	44.8	Ref.	
BPV Tertile 2	2330	740	47.2	0.99 (0.89, 1.10)	0.842	2917	954	50.2	1.10 (1.00, 1.21)	0.040
BPV Tertile 3	2305	800	55.2	1.17 (1.06, 1.30)	0.002	2924	1030	55.2	1.17 (1.07, 1.28)	p < 0.001

Adjusted for age, ethnicity, education, depression, diabetes, dyslipidemia, BMI, smoking, alcohol, baseline 3MS, living situation, average systolic blood pressure (Baseline-Year 2), antihypertensive medications.

### CIND

Similar to dementia, the occurrence of CIND increased with increasing BPV tertile in both males and females during a median follow-up of 7.3 years after the BPV estimation period, from a low of 700 events in males in tertile 1 to a high of 800 events for those in tertile 3 (Fig. 2). In females, events increased from 897 in BPV tertile 1 to 1 030 in BPV tertile 3 (Table 2). When BPV was examined as a continuous variable, the HR for CIND in the fully adjusted model in males was 1.06 (95% CI = 1.03, 1.10; p < 0.001) and 1.05 (95% CI = 1.02, 1.08; p = 0.001) in females. Participants in the highest SD tertile of BPV in both sexes also had similar elevated risk for CIND (males: HR = 1.17, 95% CI = 1.06, 1.30; p = 0.002; females: HR = 1.17, 95% CI = 1.07, 1.28; p < 0.001), compared to the lowest BPV tertile.

### Sensitivity analyses for incident dementia and CIND

When the BPV estimation period was expanded to include four BP timepoints (baseline, annual study visit Year 1, 2 and 3), and with follow-up for outcomes commencing after annual visit Year 3, the results were congruent with the primary analysis (Supplemental Table 1). The exception was the risk of CIND for females in the highest SD tertile relative to the lowest, which lost significance in the fully adjusted model (HR = 1.06, 95% CI = 0.96, 1.17; p = 0.254); however, when BPV was examined as a continuous variable, the risk remained significantly increased (HR = 1.05, 95% CI = 1.01, 1.08; p = 0.017). Likewise, when SD was replaced with ARV for the estimation of BPV, results followed a similar pattern as the main findings, with the additional finding that the risk of dementia in females in tertile 3 compared to tertile 1 was no longer significant (HR = 1.18, 95% CI = 0.99, 1.41; p = 0.063) (Supplemental Table 2).

### Subgroup analysis of ApoE-genotyped participants

The demographics of the ApoE-genotyped subgroup of participants for the incident dementia analysis are shown in Supplemental Table 3. When stratified by presence or absence of the ɛ4 allele, participants were similar in most characteristics. However, compared to those without the ɛ4 allele, there was a higher incidence of dementia (16.1/1000 person years vs 6.87/1000 person years) and cognitive decline (64.5/1000 person years vs 42.9 person years) in those with the ɛ4 allele (Supplemental Figure). When the Cox proportional hazards analysis was repeated for the association between BPV and incident dementia and CIND, stratified by presence or absence of ApoE ɛ4 allele (Table 3), the risk of dementia in males was no longer increased with high BPV; however, a significantly increased risk for CIND remained for males in the highest tertile compared to the lowest tertile for those without the ɛ4 allele (HR = 1.25, 95% CI = 1.09, 1.43; p = 0.001). In contrast, dementia risk in females without the ɛ4 allele was increased for those in the highest tertile compared to the lowest (HR = 1.39, 95% CI = 1.04, 1.84; p = 0.024), and similarly, their risk of CIND was also increased in those with, and without, the ɛ4 allele (ɛ4 allele present, HR = 1.25, 95% CI = 1.04, 1.50; p = 0.018; ɛ4 allele absent, HR = 1.14, 95% CI = 1.01, 1.29; p = 0.041).

**Table 3:** Cox proportional hazards analysis for the association between systolic blood pressure variability (BPV) and incident dementia and cognitive decline (CIND), according to sex and ApoE ɛ4 genotype.

	Males					Females				
	N	# of events	Event Rate per 1000 person years	HR (95% CI)	p-value	N	# of events	Event Rate per 1000 person years	HR (95% CI)	p-value
**Dementia**										
***ε4 allele present***	1610	226	18.7			1832	250	17.5		
BPV (continuous, per 5 mmHg)				1.07 (0.95, 1.20)	0.279				1.04 (0.94, 1.16)	0.420
BPV Tertile 1	556	66	15.7	Ref.		613	82	17.1	Ref.	
BPV Tertile 2	520	77	19.4	1.23 (0.88, 1.71)	0.229	608	84	17.8	0.98 (0.72, 1.34)	0.908
BPV Tertile 3	534	83	21.1	1.33 (0.95, 1.85)	0.094	611	84	17.8	1.10 (0.80, 1.50)	0.568
***ε4 allele absent***	4482	319	9.1			5501	321	7.2		
BPV (continuous, per 5 mmHg)				1.06 (0.97, 1.17)	0.215				1.08 (0.99, 1.18)	0.089
BPV Tertile 1	1544	101	8.2	Ref.		1841	86	5.7	Ref.	
BPV Tertile 2	1444	96	8.4	0.90 (0.68, 1.19)	0.462	1836	114	7.7	1.35 (1.02, 1.79)	0.035
BPV Tertile 3	1494	122	10.7	1.19 (0.91, 1.56)	0.198	1824	121	8.3	1.39 (1.04, 1.84)	0.024
**Cognitive decline (CIND)**										
***ε4 allele present***	1544	613	62.1			1747	735	65.2		
BPV (continuous, per 5 mmHg)				1.02 (0.95, 1.10)	0.560				1.09 (1.03, 1.15)	0.005
BPV Tertile 1	515	198	60.0	Ref.		586	226	58.5	Ref.	
BPV Tertile 2	515	207	61.0	0.96 (0.79, 1.17)	0.666	582	243	65.4	1.08 (0.90, 1.30)	0.426
BPV Tertile 3	514	208	65.6	1.06 (0.86, 1.29)	0.598	579	266	72.1	1.25 (1.04, 1.50)	0.018
***ε4 allele absent***	4312	1275	43.6			5328	1583	43.5		
BPV (continuous, per 5 mmHg)				1.08 (1.03, 1.13)	0.001				1.04 (1.00, 1.09)	0.038
BPV Tertile 1	1453	390	38.9	Ref.		1791	497	39.4	Ref.	
BPV Tertile 2	1426	410	41.4	0.99 (0.86, 1.14)	0.911	1776	515	43.0	1.08 (0.95, 1.22)	0.234
BPV Tertile 3	1433	475	50.8	1.25 (1.09, 1.43)	0.001	1761	571	48.5	1.14 (1.01, 1.29)	0.041

Adjusted for age, ethnicity, education, depression, diabetes, dyslipidemia, BMI, smoking, alcohol, baseline 3MS, living situation, average systolic blood pressure (Baseline-Year 2), antihypertensive medications. ɛ4 allele presence was defined as having ≥1 ApoE ɛ4 allele (e.g., ApoE ɛ3/ɛ4, ApoE ɛ4/ɛ4, ApoE ɛ1/ɛ4, ApoE ɛ1/ɛ3:ɛ2/ɛ4).

### Trajectories of individual cognitive tests

Finally, linear mixed models for the association between BPV and individual cognitive tests are shown in Supplemental Tables 4–7. For 3MS, results were similar in both sexes with higher BPV associated with both a lower 3MS score and an increased rate of 3MS decline over time (interaction term −0.069 for males and −0.082 for females tertile 3 vs. tertile 1, p < 0.01). For HVLT-R a similar pattern of increased decline over time for those with higher BPV was observed (interaction term −0.026 for males and −0.021 for females tertile 3 vs. tertile 1, p < 0.01), although the cross-sectional association at baseline was not statistically significant. For SDMT, higher BPV was associated with an increased rate of decline in females only (interaction term −0.52, p = 0.04 for females; −0.037, p = 0.17 for males). There was no significant association between BPV and COWAT scores at baseline or change in COWAT scores over time in either sex.

## Discussion

In this analysis of more than 15 000 initially cognitively intact, community-dwelling older adults, we found that higher visit-to-visit systolic BPV in late-life was associated with long-term increased risk of incident dementia and CIND in males, and CIND in females, independent of mean BP. Trajectory analysis of individual cognitive tests revealed similar findings in both sexes with higher BPV associated with increased rates of decline in 3MS and HVLT over time and in SDMT in females. In addition to varying by sex, the association of BPV with dementia and CIND differed by ApoE ɛ4 carrier status. Specifically, the association of BPV with both dementia and CIND was attenuated in male ApoE ɛ4 carriers. However, high systolic BPV was associated with increased risk of CIND in males lacking the ɛ4 allele, and both dementia and CIND were increased in females lacking the ɛ4 allele, suggesting high BPV in late-life is an accelerator of deteriorating long-term cognition, particularly in the absence of ApoE ɛ4.

In recent years, high systolic BPV has gained recognition as a risk factor for the development of dementia and cognitive impairment with aging [11]. A 2021 meta-analysis of 20 studies (mean participant age: 73 years) showed that high systolic BPV (independent of mean BP) increased the risk of dementia and cognitive impairment, but the GRADE quality of evidence rating was very low due to imprecision, inconsistency, and evidence of publication bias [31]. Our results add to this growing body of literature supporting late-life BPV as an independent risk factor for long-term development of dementia and cognitive decline in initially cognitively intact older adults, and also reveal new understanding that the relationship is partially influenced by sex and ApoE ɛ4 carrier status. Although BPV is not routinely monitored or screened in clinical practice, it reveals important risk information independent of mean BP; future research is needed to standardize the methodology for assessment and ultimately define whether it should be a specific treatment target [32].

The underlying mechanisms for how high BPV adversely affects cognition and differences across the sexes are not fully understood. High BPV has been proposed to be a potential marker of fluctuating hypertensive and hypotensive episodes, which could result from aging-related impairments in baroreflex sensitivity and cerebral autoregulation [33]. This dysregulation could increase vulnerability to cerebral hypoperfusion and its effects on cognition. In the SPRINT trial, recurrent hypotensive episodes captured on 24-hour ambulatory BP monitoring were associated with lower cognitive scores and faster decline in digit symbol coding even after adjustment for 24-hour average BP and variability [34]. Other postulated mechanisms for BPV’s impact on cognition include increased endothelial dysfunction and inflammation, cerebral small vessel disease, increased arterial stiffness, and amyloid-beta deposits [11]. We previously investigated the association between long-term BPV and regional cortical thickness on brain MRI, and found that increased BPV was associated with reduced cortical thickness (a measure of atrophy) in temporal, parietal, and postural frontal areas [35].

Sex is an important biological variable in aging, but few prior studies of BPV and cognition in older populations have examined the risks separately in males and females. Apart from our own research, analyses from the Korean National Health Insurance System (KNHIS) cohort [36] and the Chicago Health and Aging Project (CHAP) [37] both reported stronger associations between systolic BPV and dementia (KNHIS) and cognitive decline (CHAP) in males compared to females. These sex differences may reflect underlying biological variations in the mechanisms of dementia and cognitive decline, such as vascular changes that may mediate associations between increased BPV and cognition. Vascular dementia is more common in males than in females, and the impact and timing of exposure to specific risk factors for vascular dementia, such as lifetime alcohol use and smoking, can differ by sex. For example, an analysis of the Personality and Total Health Through Life Project found that incident midlife hypertension was associated with greater subsequent declines in memory in females compared to males, while later-life stroke was associated with greater declines in memory in males compared to females [38]. Future work is needed to better understand the physiologic mechanisms underlying the observed sex-based differences in the impact of BPV on cognition [11].

Our findings incorporating ApoE genotype reveal complexity in the association between late-life visit-to-visit BPV and long-term cognitive change. The ApoE gene, particularly the ɛ4 allele, is the strongest genetic determinant of all-cause dementia [39]. The association between high BPV and risk of dementia or CIND in males was attenuated in those carrying the APOE ɛ4 allele; however, males lacking the ɛ4 allele remained at increased risk of CIND. In females, high BPV was significantly associated with increased risk of dementia when the ɛ4 allele was absent, and increased risk of CIND regardless of ɛ4 carrier status. In all fully adjusted models comparing risk in the highest tertile of BPV to the lowest, the increased hazard ratios observed in the analysis of the full cohort were preserved for both sexes in the ApoE-genotyped cohort, suggesting that loss of statistical significance could reflect inadequate power since ApoE genotype was available for only 81% of the cohort. Alternate explanations for the differences in BPV-associated risk of dementia and cognitive decline based on sex and ApoE genotype include the possibility that the underlying pathologic mechanisms of dementia and cognitive decline in individuals with and without the ɛ4 allele differ; for example, dementia in those lacking an ɛ4 allele could be primarily vascular-related and contributed by age-related dynamic instability in BP, while individuals with the ɛ4 allele may have more Alzheimer’s disease pathology less influenced by BPV. Other research in Alzheimer’s Disease Neuroimaging Initiative participants has reported accelerated cognitive decline and increased medial temporal volume loss with high BPV in ApoE ɛ4 carriers, but did not examine sex-based differences [40, 41]. ApoE may have an association with midlife vascular mortality in males that could contribute to the observed sex differences in associations between ApoE and dementia in late life [42]. Caution has been advised in interpreting sex-specific genetic associations, given the differential sex survival distributions and the possibility that the ApoE gene may have pleiotropic effects influencing both the risk of dementia and mortality/longevity which can introduce spurious associations [43].

Key strengths of our study include the large sample size, a priori analyses by sex, robust covariate adjustment, stratification by ApoE carrier status, and longitudinal standardized measures of BP and cognition. Additional strengths include adjudication of the dementia endpoint and nearly a decade of follow-up for endpoints occurring after the BPV estimation period. The findings from the ASPREE cohort are generalizable to a large proportion of older community-dwelling adults who have reached late-life while cognitively intact. Additional factors increasing the generalizability of our findings include that not all participants were hypertensive, those with severe uncontrolled BP were excluded from ASPREE, and the management of BP in the study was left to the discretion of the participant’s primary care provider; thus, the risk estimates for BPV could be higher in a general population with greater hypertensive burden.

We also acknowledge important limitations of our study, including its post-hoc, exploratory nature, the possibility of unmeasured confounders, and potential for ascertainment/healthy survivorship bias, with only individuals without a prior diagnosis of cardiovascular disease or other life-threating illness enrolling. Participants in ASPREE were mostly healthy as a condition of enrollment into the trial, which does limit the generalizability of our findings in populations with greater morbidity burden or who already have subclinical cognitive impairment. Due to the timing of collection of concomitant medications (yearly at annual study visits), we did not account for interim initiation of specific antihypertensives which can affect BPV; however, we controlled for baseline antihypertensive use and mean BP, and the overall annual rate of change in antihypertensive use (discontinuation, switching, or new starts) during the BPV estimation period was very low (approximately 2%). Although we used a two-year period to characterize long-term BPV of the participants, our findings were consistent when we expanded to three years, and the optimal duration necessary to adequately reflect long-term BPV is not currently known [32]. Our definition of cognitive impairment was study-specific and may have overestimated the true incidence of cognitive impairment; however, the point estimates were directionally consistent with the more rigorous adjudicated dementia endpoint. Lastly, our findings are associative only, and could reflect reverse causality if autonomic dysfunction from neurodegeneration is an underlying pathophysiologic mechanism contributing to increased BPV.

## Conclusion

High visit-to-visit systolic BPV in late-life was independently associated with increased long-term risks of dementia and cognitive decline in males, and cognitive decline in females. In ApoE-genotyped participants, high BPV was associated with dementia in females and cognitive decline in males when the ɛ4 allele was absent, and cognitive decline in females regardless of ɛ4 carrier status, suggesting a mechanism of risk partially independent to the effects of ApoE ɛ4. Our findings indicate that both sex and the presence of ApoE ɛ4 allele influence the associations between late-life visit-to-visit BPV and long-term risks of dementia and cognitive decline in older adults. Additional research is necessary to understand the mechanism of this relationship, and inform the optimal BP management strategies to help preserve cognition in adults reaching older ages cognitively intact.

## Summary

### What is known about the topic?

- High blood pressure variability has been previously shown to predict increased risks for dementia and cognitive decline.
- Sex-based differences, and the interplay of apolipoprotein E, with high late-life, visit-to-visit systolic blood pressure variability on the long-term risks of dementia and cognitive decline in cognitively intact older adults remains largely unexplored.

### What this study adds?

- Using extended follow-up from the ASPREE study, we found that high late-life, visit-to-visit BPV is associated with increased risk of dementia in males, and cognitive decline in both males and females.
- The presence, or absence, of the ApoE ɛ4 allele influences sex-based differences in the risk dementia and cognitive decline observed with high visit-to-visit variability in systolic blood pressure.

## Supplementary information

Supplemental Material

## Data Availability

The data that support the findings of this study are available from the ASPREE Data Coordinating Center, Monash University School of Public Health (https://ams.aspree.org/public/) upon reasonable request. More information is available at: https://ams.aspree.org/public/.
