# Supplementary material for: Late-life, visit-to-visit blood pressure variability and its association with sex-specific long-term cognitive outcomes
Source: J Hum Hypertens. 2025 Oct 18;39(12):831–40. doi: 10.1038/s41371-025-01087-5 (PMC12685738; doi:10.1038/s41371-025-01087-5)
Supplement: Supplementary file 1 — Supplemental Material [file 41371_2025_1087_MOESM1_ESM.docx]

**Late-life, visit-to-visit blood pressure variability and its association with sex-specific long-term cognitive outcomes**

Michael E. Ernst, PharmD; Kerry Sheets, MD, MS; Katherine L. Webb, MA; Michelle A. Fravel, PharmD; Robyn L. Woods, PhD; Lawrence Beilin, MB, MD; Suzanne G. Orchard, PhD; Raj C. Shah, MD; Kevan Polkinghorne, PhD; Christopher M. Reid, PhD; Rory Wolfe, PhD; Anne Murray, MD; Paul Lacaze, PhD; Joanne Ryan, PhD

**Supplemental Tables.**

**Table 1.** Cox proportional hazards analysis for the association between systolic blood pressure variability (BPV), calculated using baseline, year 1, 2, and 3 visits, and incident dementia and cognitive decline (CIND).

**Table 2.** Cox proportional hazards analysis for the association between systolic blood pressure variability (BPV), calculated using absolute real variability, and incident dementia and cognitive decline (CIND).

**Table 3.** Baseline characteristics of ASPREE participants included in the incident dementia analysis, stratified by presence or absence of ApoE ɛ4 allele.

**Table 4.**  Longitudinal changes in 3MS score according to standard deviation tertiles (T) of systolic blood pressure variability, by sex.

**Table 5.** Longitudinal changes in COWAT score according to standard deviation tertiles (T) of systolic blood pressure variability, by sex.

**Table 6.** Longitudinal changes in HVLT-R score according to standard deviation tertiles (T) of systolic blood pressure variability, by sex.

**Table 7.** Longitudinal changes in SDMT score according to standard deviation tertiles (T) of systolic blood pressure variability, by sex.

**Supplemental Figure.**

**Panel A.** Cumulative incidence of adjudicated dementia events according to standard deviation tertiles of systolic blood pressure variability and ApoE ɛ4 allele presence.

**Panel B.** Cumulative incidence of cognitive decline (CIND) according to standard deviation tertiles of systolic blood pressure variability and ApoE ɛ4 allele presence.

**Supplemental Table 1.** Cox proportional hazards analysis for the association between systolic blood pressure variability (BPV), calculated using baseline, year 1, 2, and 3 visits, and incident dementia and cognitive decline (CIND).

|  |  |  | **Model 1** | | **Model 2** | | **Model 3** | |
| --- | --- | --- | --- | --- | --- | --- | --- | --- |
|  | **N** | **# of events** | **HR (95% CI)** | **p-value** | **HR (95% CI)** | **p-value** | **HR (95% CI)** | **p-value** |
| **Dementia** |  |  |  |  |  |  |  |  |
| **Males** | 6802 | 570 |  |  |  |  |  |  |
| BPV (continuous, per 5mmHg) |  |  | 1.11 (1.03, 1.20) | 0.008 | 1.09 (1.01, 1.18) | 0.028 | 1.10 (1.02, 1.20) | 0.014 |
| BPV Tertile 1 | 2269 | 158 | Ref. |  | Ref. |  | Ref. |  |
| BPV Tertile 2 | 2266 | 186 | 1.14 (0.92, 1.41) | 0.237 | 1.10 (0.89, 1.36) | 0.394 | 1.11 (0.89, 1.37) | 0.356 |
| BPV Tertile 3 | 2267 | 226 | 1.47 (1.20, 1.80) | p < 0.001 | 1.42 (1.16, 1.74) | p < 0.001 | 1.46 (1.19, 1.80) | p < 0.001 |
| **Females** | 8585 | 627 |  |  |  |  |  |  |
| BPV (continuous, per 5mmHg) |  |  | 1.05 (0.98, 1.12) | 0.199 | 1.04 (0.97, 1.11) | 0.285 | 1.04 (0.97, 1.11) | 0.310 |
| BPV Tertile 1 | 2872 | 185 | Ref. |  | Ref. |  | Ref. |  |
| BPV Tertile 2 | 2856 | 213 | 1.12 (0.92, 1.37) | 0.253 | 1.12 (0.92, 1.36) | 0.276 | 1.12 (0.92, 1.36) | 0.279 |
| BPV Tertile 3 | 2857 | 229 | 1.20 (0.99, 1.46) | 0.065 | 1.18 (0.97, 1.44) | 0.092 | 1.18 (0.97, 1.44) | 0.099 |
| **Cognitive decline (CIND)** |  |  |  |  |  |  |  |  |
| **Males** | 6176 | 1912 |  |  |  |  |  |  |
| BPV (continuous, per 5mmHg) |  |  | 1.10 (1.05, 1.14) | p < 0.001 | 1.08 (1.04, 1.13) | p < 0.001 | 1.08 (1.03, 1.13) | p < 0.001 |
| BPV Tertile 1 | 2060 | 576 | Ref. |  | Ref. |  | Ref. |  |
| BPV Tertile 2 | 2064 | 636 | 1.06 (0.94, 1.18) | 0.330 | 1.04 (0.93, 1.16) | 0.540 | 1.03 (0.92, 1.16) | 0.554 |
| BPV Tertile 3 | 2052 | 700 | 1.30 (1.16, 1.45) | p < 0.001 | 1.26 (1.13, 1.41) | p < 0.001 | 1.26 (1.12, 1.41) | p < 0.001 |
| **Females** | 7798 | 2510 |  |  |  |  |  |  |
| BPV (continuous, per 5mmHg) |  |  | 1.06 (1.02, 1.10) | 0.002 | 1.05 (1.02, 1.09) | 0.003 | 1.05 (1.01, 1.08) | 0.017 |
| BPV Tertile 1 | 2601 | 792 | Ref. |  | Ref. |  | Ref. |  |
| BPV Tertile 2 | 2600 | 832 | 1.03 (0.93, 1.13) | 0.609 | 1.02 (0.93, 1.13) | 0.644 | 1.01 (0.92, 1.12) | 0.780 |
| BPV Tertile 3 | 2597 | 886 | 1.09 (0.99, 1.20) | 0.069 | 1.08 (0.99, 1.19) | 0.098 | 1.06 (0.96, 1.17) | 0.254 |

Model 1: BPV + age

Model 2: Model 1 + ethnicity, education, depression, diabetes, dyslipidemia, BMI, smoking, alcohol, baseline 3MS, living situation

Model 3: Model 2 + average systolic blood pressure (Baseline-Year 2), antihypertensive medications

**Supplemental Table 2.** Cox proportional hazards analysis for the association between systolic blood pressure variability (BPV), calculated using absolute real variability, and incident dementia and cognitive decline (CIND).

|  |  |  | **Model 1** | | **Model 2** | | **Model 3** | |
| --- | --- | --- | --- | --- | --- | --- | --- | --- |
|  | **N** | **# of events** | **HR (95% CI)** | **p-value** | **HR (95% CI)** | **p-value** | **HR (95% CI)** | **p-value** |
| **Dementia** |  |  |  |  |  |  |  |  |
| **Males** | 7335 | 660 |  |  |  |  |  |  |
| BPV (continuous, per 5mmHg) |  |  | 1.07 (1.02, 1.12) | 0.007 | 1.06 (1.01, 1.11) | 0.020 | 1.06 (1.01, 1.11) | 0.012 |
| BPV Tertile 1 | 2632 | 207 | Ref. |  | Ref. |  | Ref. |  |
| BPV Tertile 2 | 2333 | 191 | 1.02 (0.84, 1.25) | 0.821 | 1.00 (0.83, 1.22) | 0.963 | 1.01 (0.83, 1.23) | 0.914 |
| BPV Tertile 3 | 2370 | 262 | 1.41 (1.18, 1.70) | p < 0.001 | 1.37 (1.14, 1.65) | p < 0.001 | 1.40 (1.17, 1.69) | p < 0.001 |
| **Females** | 9294 | 739 |  |  |  |  |  |  |
| BPV (continuous, per 5mmHg) |  |  | 1.03 (0.99, 1.07) | 0.207 | 1.02 (0.98, 1.06) | 0.336 | 1.02 (0.98, 1.06) | 0.340 |
| BPV Tertile 1 | 3328 | 243 | Ref. |  | Ref. |  | Ref. |  |
| BPV Tertile 2 | 3008 | 232 | 1.07 (0.89, 1.28) | 0.476 | 1.06 (0.88, 1.26) | 0.551 | 1.06 (0.88, 1.27) | 0.537 |
| BPV Tertile 3 | 2958 | 264 | 1.18 (0.99, 1.41) | 0.058 | 1.18 (0.99, 1.41) | 0.063 | 1.18 (0.99, 1.41) | 0.063 |
| **Cognitive decline (CIND)** |  |  |  |  |  |  |  |  |
| **Males** | 6961 | 2240 |  |  |  |  |  |  |
| BPV (continuous, per 5mmHg) |  |  | 1.05 (1.02, 1.08) | p < 0.001 | 1.04 (1.02, 1.07) | 0.001 | 1.04 (1.01, 1.07) | 0.003 |
| BPV Tertile 1 | 2528 | 760 | Ref. |  | Ref. |  | Ref. |  |
| BPV Tertile 2 | 2207 | 701 | 1.04 (0.94, 1.15) | 0.462 | 1.04 (0.94, 1.15) | 0.464 | 1.04 (0.94, 1.15) | 0.473 |
| BPV Tertile 3 | 2226 | 779 | 1.21 (1.10, 1.34) | p < 0.001 | 1.20 (1.08, 1.32) | p < 0.001 | 1.19 (1.07, 1.32) | p < 0.001 |
| **Females** | 8816 | 2881 |  |  |  |  |  |  |
| BPV (continuous, per 5mmHg) |  |  | 1.03 (1.01, 1.06) | 0.002 | 1.03 (1.01, 1.05) | 0.003 | 1.03 (1.01, 1.05) | 0.015 |
| BPV Tertile 1 | 3181 | 972 | Ref. |  | Ref. |  | Ref. |  |
| BPV Tertile 2 | 2845 | 934 | 1.12 (1.02, 1.23) | 0.013 | 1.11 (1.02, 1.22) | 0.020 | 1.10 (1.01, 1.21) | 0.030 |
| BPV Tertile 3 | 2790 | 975 | 1.18 (1.08, 1.29) | p < 0.001 | 1.17 (1.07, 1.28) | p < 0.001 | 1.15 (1.05, 1.26) | 0.002 |

Model 1: BPV + age

Model 2: Model 1 + ethnicity, education, depression, diabetes, dyslipidemia, BMI, smoking, alcohol, baseline 3MS, living situation

Model 3: Model 2 + average systolic blood pressure (Baseline-Year 2), antihypertensive medications

**Supplemental Table 3.** Baseline characteristics of ASPREE participants included in the incident dementia analysis, stratified by presence or absence of ApoE ɛ4 allele.

|  | ε4 allele present, n = 3,442 | ε4 allele absent, n = 9,983 |
| --- | --- | --- |
| **Number of dementia events (rate/1000py)** | 345 (16.10) | 441 (6.87) |
| **Number of cognitive decline (CIND) events (rate/1000py)** | 1144 (64.46) | 2344 (42.93) |
| **Standard deviation of SBP, mean (SD)** | 10.07 (6.02) | 10.07 (5.94) |
| **Standard deviation of SBP, range** | 0-46.69 | 0-50.08 |
| **Baseline SBP, mmHg, mean (SD)** | 139.18 (16.32) | 139.07 (16.33) |
| **Baseline DBP, mmHg, mean (SD)** | 77.38 (9.95) | 77.08 (9.92) |
| **Baseline antihypertensive medications, n (%)** | 1708 (49.6) | 5153 (51.6) |
| **Average SBP over BPV period, mean (SD)** | 137.82 (13.60) | 137.81 (13.70) |
| **Sex, female, n (%)** | 1832 (53.2) | 5501 (55.1) |
| **Ethno-racial group, n (%)** |  |  |
| Australian white | 3093 (89.9) | 9040 (90.6) |
| U.S. white | 158 (4.6) | 500 (5.0) |
| African-American | 123 (3.6) | 204 (2.0) |
| Hispanic/Latino | 33 (1.0) | 104 (1.0) |
| Other | 35 (1.0) | 135 (1.4) |
| **Age, y, n (%)** |  |  |
| 65-73 | 1895 (55.1) | 5017 (50.3) |
| ≥ 74 | 1547 (44.9) | 4966 (49.7) |
| **Education y, n (%)** |  |  |
| <12 | 1555 (45.2) | 4512 (45.2) |
| 12+ | 1887 (54.8) | 5471 (54.8) |
| **Alcohol use, n (%)** |  |  |
| Current | 2672 (77.6) | 7946 (79.6) |
| Former | 187 (5.4) | 494 (4.9) |
| Never | 583 (16.9) | 1543 (15.5) |
| **Body Mass Index, kg/m^2^, mean (SD)** | 27.80 (4.53) | 28.09 (4.56) |
| **Living alone, n (%)** | 1072 (31.1) | 3152 (31.6) |
| **Current or past smoker, n (%)** | 1547 (44.9) | 4357 (43.6) |
| **Diabetes, n (%)** | 296 (8.6) | 999 (10.0) |
| **Depression, n (%)** | 324 (9.4) | 897 (9.0) |
| **Dyslipidemia, n (%)** | 2518 (73.2) | 6307 (63.2) |
| **Chronic Kidney Disease, n (%)** | 790 (24.7) | 2356 (25.4) |
| **Hypertension, n (%)** | 2487 (72.3) | 7366 (73.8) |
| **Statin medications, n (%)** | 1284 (37.3) | 2850 (28.5) |
| **Anti-hypertension medications, n (%)**  (not mutually exclusive) |  |  |
| ACE inhibitors | 579 (16.8) | 1701 (17.0) |
| ARBs | 862 (25.0) | 2684 (26.9) |
| CCBs | 588 (17.1) | 1748 (17.5) |
| Diuretics | 629 (18.3) | 1844 (18.5) |
| **Aspirin treatment assignment, n (%)** | 1737 (50.5) | 4939 (49.5) |
| **Pulse Pressure, mean (SD)** | 61.80 (13.33) | 61.98 (13.75) |
| **Heart Rate, mean (SD)** | 70.27 (10.65) | 70.76 (10.50) |
| **Standard deviation of heart rate variability, mean (SD)** | 5.48 (3.75) | 5.46 (3.70) |
| **Cognitive performance** |  |  |
| 3MS, mean (SD) | 93.64 (4.46) | 93.88 (4.32) |
| HVLT-R delayed recall, mean (SD) | 7.78 (2.81) | 7.96 (2.75) |
| SDMT, mean (SD) | 37.16 (9.94) | 37.70 (9.81) |
| COWAT, mean (SD) | 12.51 (4.56) | 12.17 (4.56) |

SBP = systolic blood pressure. DBP = diastolic blood pressure. Diabetes defined as a self-report, fasting glucose ≥126 mg/dl, or receiving pharmacologic treatment for diabetes (regardless of fasting glucose level). Depressive symptoms defined as a 10-item Center for Epidemiological Studies Depression Scale^23^ score of ≥8. Dyslipidemia defined as serum cholesterol level of ≥212 mg per deciliter (≥5.5 mmol per liter) in Australia and ≥240 mg per deciliter (≥6.2 mmol per liter) in the United States, low-density lipoprotein level of >160 mg per deciliter (>4.1 mmol per liter), or use of a cholesterol-lowering medication. Chronic kidney disease defined as estimated glomerular filtration rate <60 mL/min per 1.73 m2 or albumin to creatinine ratio ≥3 mg/mmol. Hypertension defined as SBP ≥140 mmHg, DBP ≥90 mmHg, or receiving treatment for high BP (regardless of BP level). 3MS = modified mini-mental state examination.^19^ HVLT-R = Hopkins Verbal Learning Test-Revised.^20^ SDMT = Symbol Digit Modalities Test.^22^ COMT = Controlled Oral Word Association Test.^21^

**Supplemental Table 4.**  Longitudinal changes in 3MS score according to standard deviation tertiles (T) of systolic blood pressure variability, by sex.

|  | **Continuous BPV (per 5 mmHg)** | | **T1** | **T2** | | **T3** | |
| --- | --- | --- | --- | --- | --- | --- | --- |
| **Males** |  |  |  |  |  |  |  |
|  | Estimate¹ | Interaction² |  | Estimate¹ | Interaction² | Estimate¹ | Interaction² |
| Model 1 | -0.246  (p < 0.01) | -0.022  (p < 0.01) | Ref. | -0.230  (p = 0.22) | -0.041  (p = 0.05) | -0.509  (p < 0.01) | -0.072  (p < 0.01) |
| Model 2 | -0.206  (p < 0.01) | -0.021  (p < 0.01) | Ref. | -0.228  (p = 0.22) | -0.039  (p = 0.06) | -0.446  (p = 0.02) | -0.069  (p < 0.01) |
| Model 3 | -0.183  (p < 0.01) | -0.021  (p < 0.01) | Ref. | -0.214  (p = 0.25) | -0.039  (p = 0.07) | -0.386  (p = 0.04) | -0.069  (p < 0.01) |
| **Females** |  |  |  |  |  |  |  |
|  | Estimate¹ | Interaction² |  | Estimate¹ | Interaction² | Estimate¹ | Interaction² |
| Model 1 | -0.075  (p = 0.18) | -0.029  (p < 0.01) | Ref. | 0.165  (p = 0.32) | -0.074  (p < 0.01) | -0.076  (p = 0.65) | -0.082  (p < 0.01) |
| Model 2 | -0.043  (p = 0.43) | -0.029  (p < 0.01) | Ref. | 0.122  (p = 0.46) | -0.072  (p < 0.01) | -0.061  (p = 0.71) | -0.082  (p < 0.01) |
| Model 3 | -0.015  (p = 0.79) | -0.029  (p < 0.01) | Ref. | 0.155  (p = 0.35) | -0.072  (p < 0.01) | 0.019  (p = 0.91) | -0.082  (p < 0.01) |

¹Estimate of the cross-sectional associated of BPV with 3MS score at baseline. Note, excludes 17 measurements at annual visit 2 as 3MS was not scheduled for collection at annual visit 2.

²Time-BPV interaction term to test variation of the 3MS score trajectory among BPV levels.

Model 1: BPV + age

Model 2: Model 1 + ethnicity, education, depression, diabetes, dyslipidemia, BMI, smoking, alcohol, living situation

Model 3: Model 2 + average systolic blood pressure (Baseline-Year 2), antihypertensive medications

**Supplemental Table 5.** Longitudinal changes in COWAT score according to standard deviation tertiles (T) of systolic blood pressure variability, by sex.

|  | **Continuous BPV (per 5 mmHg)** | | | **T1** | **T2** | | **T3** | |
| --- | --- | --- | --- | --- | --- | --- | --- | --- |
| **Males** |  | |  |  |  |  |  |  |
|  | Estimate¹ | | Interaction² |  | Estimate¹ | Interaction² | Estimate¹ | Interaction² |
| Model 1 | -0.079  (p = 0.11) | | -0.009  (p = 0.11) | Ref. | -0.295  (p = 0.03) | 0.027  (p = 0.08) | -0.197  (p = 0.16) | -0.023  (p = 0.13) |
| Model 2 | -0.058  (p = 0.23) | | -0.008  (p = 0.14) | Ref. | -0.315  (p = 0.02) | 0.028  (p = 0.06) | -0.180  (p = 0.18) | -0.021  (p = 0.18) |
| Model 3 | -0.051  (p = 0.30) | | -0.008  (p = 0.14) | Ref. | -0.315  (p = 0.02) | 0.028  (p = 0.06) | -0.164  (p = 0.23) | -0.021  (p = 0.18) |
| **Females** |  |  |  |  |  |  |  |  |
|  | Estimate¹ | | Interaction² |  | Estimate¹ | Interaction² | Estimate¹ | Interaction² |
| Model 1 | -0.033  (p = 0.46) | | -0.002  (p = 0.64) | Ref. | 0.161  (p = 0.22) | -0.020  (p = 0.14) | 0.068  (p = 0.60) | -0.011  (p = 0.41) |
| Model 2 | 0.007  (p = 0.87) | | -0.003  (p = 0.59) | Ref. | 0.127  (p = 0.32) | -0.019  (p = 0.17) | 0.108  (p = 0.40) | -0.012  (p = 0.39) |
| Model 3 | 0.021  (p = 0.62) | | -0.002  (p = 0.60) | Ref. | 0.148  (p = 0.25) | -0.019  (p = 0.16) | 0.148  (p = 0.25) | -0.012  (p = 0.40) |

¹Estimate of the cross-sectional associated of BPV with COWAT score at baseline.

²Time-BPV interaction term to test variation of the COWAT score trajectory among BPV levels.

Model 1: BPV + age

Model 2: Model 1 + ethnicity, education, depression, diabetes, dyslipidemia, BMI, smoking, alcohol, living situation

Model 3: Model 2 + average systolic blood pressure (Baseline-Year 2), antihypertensive medications

**Supplemental Table 6.** Longitudinal changes in HVLT-R score according to standard deviation tertiles (T) of systolic blood pressure variability, by sex.

|  | **Continuous BPV (per 5 mmHg)** | | | **T1** | **T2** | | **T3** | |
| --- | --- | --- | --- | --- | --- | --- | --- | --- |
| **Males** |  | |  |  |  |  |  |  |
|  | Estimate¹ | | Interaction² |  | Estimate¹ | Interaction² | Estimate¹ | Interaction² |
| Model 1 | -0.068  (p = 0.05) | | -0.007  (p = 0.03) | Ref. | -0.120  (p = 0.22) | 0.011  (p = 0.23) | -0.163  (p = 0.10) | -0.027  (p < 0.01) |
| Model 2 | -0.057  (p = 0.1) | | -0.007  (p = 0.03) | Ref. | -0.128  (p = 0.18) | 0.012  (p = 0.21) | -0.154  (p = 0.11) | -0.026  (p < 0.01) |
| Model 3 | -0.045  (p = 0.19) | | -0.007  (p = 0.03) | Ref. | -0.126  (p = 0.19) | 0.012  (p = 0.20) | -0.128  (p = 0.19) | -0.026  (p < 0.01) |
| **Females** |  |  |  |  |  |  |  |  |
|  | Estimate¹ | | Interaction² |  | Estimate¹ | Interaction² | Estimate¹ | Interaction² |
| Model 1 | -0.068  (p = 0.02) | | -0.004  (p = 0.20) | Ref. | 0.033  (p = 0.69) | -0.027  (p < 0.01) | -0.097  (p = 0.25) | -0.021  (p < 0.01) |
| Model 2 | -0.057  (p = 0.04) | | -0.004  (p = 0.19) | Ref. | 0.010  (p = 0.90) | -0.027  (p < 0.01) | -0.102  (p = 0.22) | -0.021  (p < 0.01) |
| Model 3 | -0.049  (p = 0.08) | | -0.004  (p = 0.19) | Ref. | 0.022  (p = 0.79) | -0.027  (p < 0.01) | -0.077  (p = 0.36) | -0.021  (p < 0.01) |

¹Estimate of the cross-sectional associated of BPV with HVLT-R score at baseline.

²Time-BPV interaction term to test variation of the HVLT-R score trajectory among BPV levels.

Model 1: BPV + age

Model 2: Model 1 + ethnicity, education, depression, diabetes, dyslipidemia, BMI, smoking, alcohol, living situation

Model 3: Model 2 + average systolic blood pressure (Baseline-Year 2), antihypertensive medications

**Supplemental Table 7.** Longitudinal changes in SDMT score according to standard deviation tertiles (T) of systolic blood pressure variability, by sex.

|  | **Continuous BPV (per 5 mmHg)** | | **T1** | **T2** | | **T3** | |
| --- | --- | --- | --- | --- | --- | --- | --- |
| **Males** |  |  |  |  |  |  |  |
|  | Estimate¹ | Interaction² |  | Estimate¹ | Interaction² | Estimate¹ | Interaction² |
| Model 1 | -0.545  (p < 0.01) | -0.013  (p = 0.18) | Ref. | -0.931  (p < 0.01) | 0.043  (p = 0.10) | -1.452  (p < 0.01) | -0.041  (p = 0.12) |
| Model 2 | -0.491  (p < 0.01) | -0.012  (p = 0.22) | Ref. | -0.978  (p < 0.01) | 0.045  (p = 0.08) | -1.412  (p < 0.01) | -0.037  (p = 0.17) |
| Model 3 | -0.453  (p < 0.01) | -0.012  (p = 0.22) | Ref. | -0.961  (p < 0.01) | 0.045  (p = 0.08) | -1.318  (p < 0.01) | -0.037  (p = 0.17) |
| **Females** |  |  |  |  |  |  |  |
|  | Estimate¹ | Interaction² |  | Estimate¹ | Interaction² | Estimate¹ | Interaction² |
| Model 1 | -0.411  (p < 0.01) | -0.014  (p = 0.10) | Ref. | 0.068  (p = 0.80) | -0.088  (p < 0.01) | -0.934  (p < 0.01) | -0.052  (p = 0.04) |
| Model 2 | -0.37  (p < 0.01) | -0.015  (p = 0.09) | Ref. | -0.024  (p = 0.93) | -0.085  (p < 0.01) | -0.938  (p < 0.01) | -0.052  (p = 0.04) |
| Model 3 | -0.267  (p < 0.01) | -0.015  (p = 0.10) | Ref. | 0.090  (p = 0.72) | -0.085  (p < 0.01) | -0.661  (p = 0.01) | -0.052  (p = 0.04) |

¹Estimate of the cross-sectional associated of BPV with SDMT score at baseline.

²Time-BPV interaction term to test variation of the SDMT score trajectory among BPV levels.

Model 1: BPV + age

Model 2: Model 1 + ethnicity, education, depression, diabetes, dyslipidemia, BMI, smoking, alcohol, living situation

Model 3: Model 2 + average systolic blood pressure (Baseline-Year 2), antihypertensive medications

**Supplemental Figure.**

**Panel A.** Cumulative incidence of adjudicated dementia events according to standard deviation tertiles of systolic blood pressure variability and ApoE ɛ4 allele presence.

**
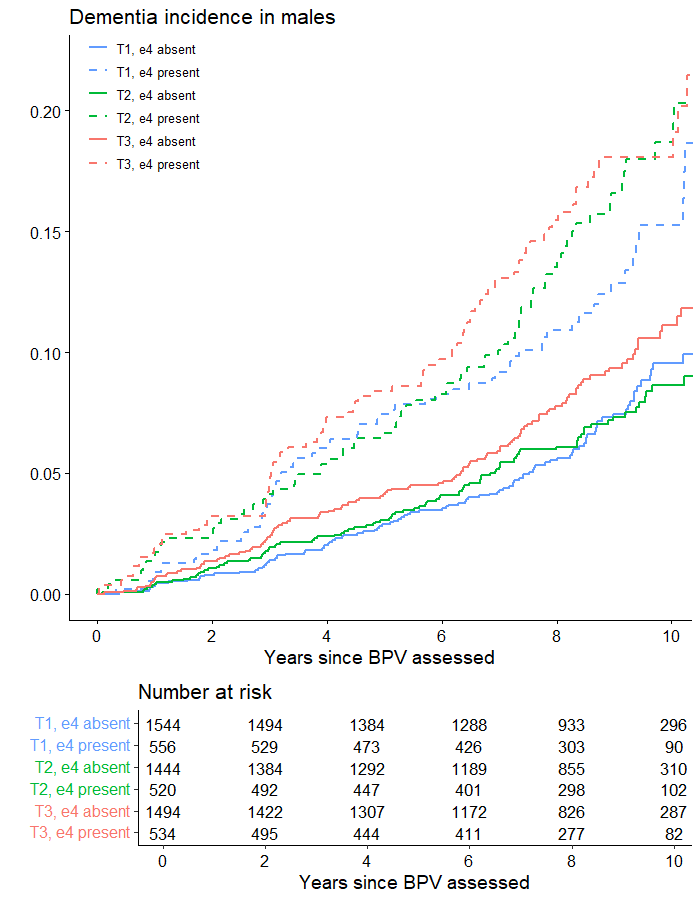

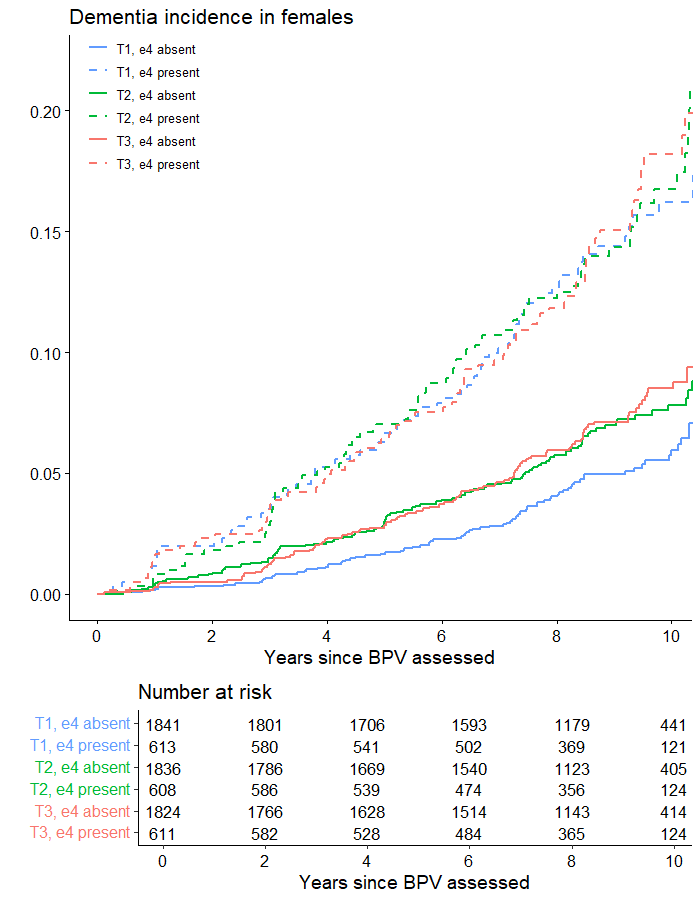
**

**Panel B.** Cumulative incidence of cognitive decline (CIND) according to standard deviation tertiles of systolic blood pressure variability and ApoE ɛ4 allele presence.

**
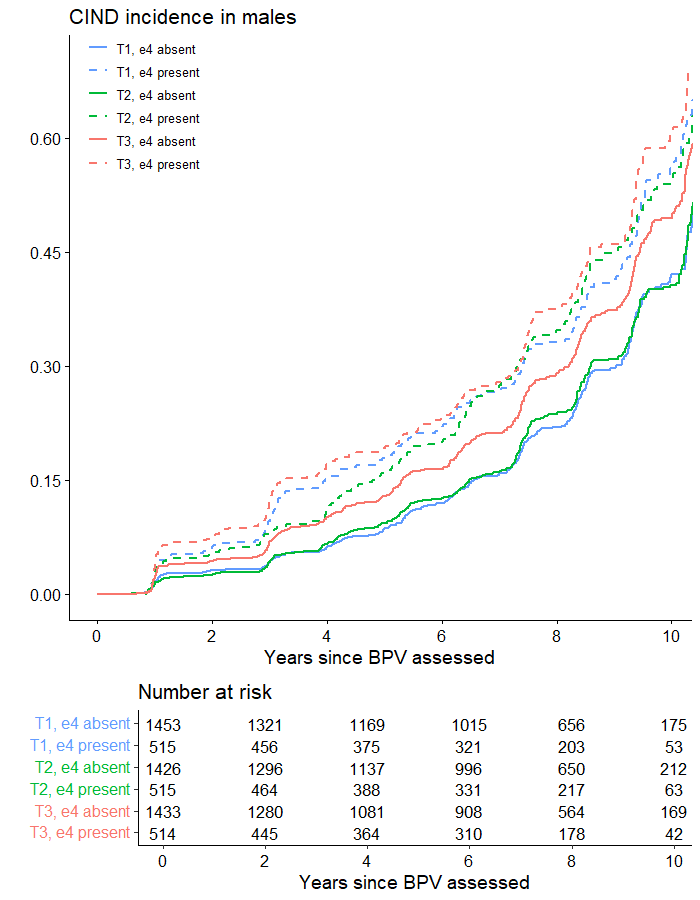

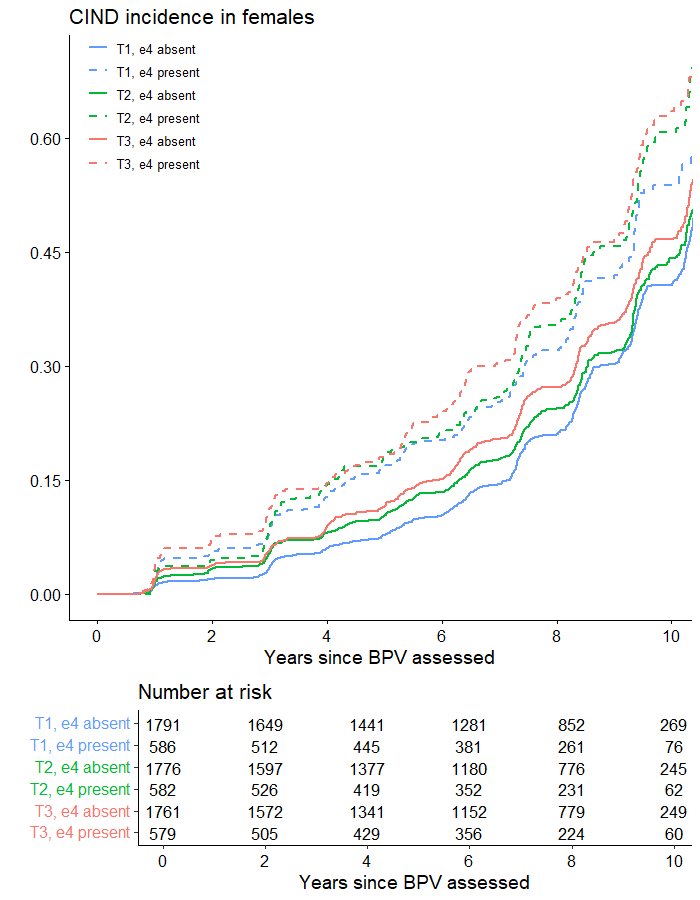
**
